# Optimizing information processing in neuronal networks beyond critical states

**DOI:** 10.1371/journal.pone.0184367

**Published:** 2017-09-18

**Authors:** Mariana Sacrini Ayres Ferraz, Hiago Lucas Cardeal Melo-Silva, Alexandre Hiroaki Kihara

**Affiliations:** Núcleo de Cognição e Sistemas Complexos, Centro de Matemática, Computação e Cognição, Universidade Federal do ABC, São Bernardo do Campo, SP, Brasil; Consejo Nacional de Investigaciones Cientificas y Tecnicas, ARGENTINA

## Abstract

Critical dynamics have been postulated as an ideal regime for neuronal networks in the brain, considering optimal dynamic range and information processing. Herein, we focused on how information entropy encoded in spatiotemporal activity patterns may vary in critical networks. We employed branching process based models to investigate how entropy can be embedded in spatiotemporal patterns. We determined that the information capacity of critical networks may vary depending on the manipulation of microscopic parameters. Specifically, the mean number of connections governed the number of spatiotemporal patterns in the networks. These findings are compatible with those of the real neuronal networks observed in specific brain circuitries, where critical behavior is necessary for the optimal dynamic range response but the uncertainty provided by high entropy as coded by spatiotemporal patterns is not required. With this, we were able to reveal that information processing can be optimized in neuronal networks beyond critical states.

## Introduction

Maximum unpredictability is reported to occur around phase transitions where criticality is observed. This hypothesis finds important concrete realizations in biological systems, including the brain physiology [1]. Although it is still under discussion, a cornerstone from the theoretical perspective is that neuronal networks work in the vicinities of a critical regime, i.e., the activities observed in the neuronal networks, *in vivo* [2,3,4,5], *in vitro* [2,6,7,8,9,10] *silico* [1,11,12,13], are found to exhibit neuronal-avalanche-like behaviors whose size distribution can be approximated by a power law.

The critical regime is known to provide some advantages for processing in network systems. In the neuronal network context, we can cite the maximization of both information processing [2,3,11] and dynamic range [1,11]. Information processing in the brain has been studied through interdisciplinary perspectives, including biological, psychophysical, and mathematical approaches [1,2,8,14,15,16]. As a theoretical example, a network of excitable elements maximizes information processing at the critical point compared with that at other conditions such as sub- and super-critical regimes [1]. The enhancement of dynamic range particularly favors neuronal networks in sensory systems [1,7]. This finding is compatible with the role of connexin-mediated communication in electrical synapses observed in the retina [17,18,19,20] and olfactory glomeruli [21,22].

Different regions of the brain, other than the sensory systems, have diverse functionalities and their activity patterns are responsible for coding and storing information. Experiments have shown that information in the cortex and hippocampus are provided through repeated spatiotemporal patterns [6,23,24,25,26,27,28] that are related to memory consolidation [24,28]. Thus, neuronal avalanches present themselves as highly diverse and also repeatable [6].

Although the pattern variability in criticality is maximum [11,13], the manner in which these patterns occur in critical networks and the way they are related to the micro-parameters of the network has not been explored thoroughly. In this study, we investigated the process of encoding information by a neuronal network in the critical regime. Furthermore, we employed simple branching process based models to demonstrate the distinct information capacity displayed by the critical networks. Our findings are compatible with those of the distinct neuronal circuitries observed in the brain, in which different information capacities are required while preserving the dynamical range provided by the critical state.

## Methods

### Network construction and avalanche statistics

The model used here is based on that reported previously [1]. Despite its simplicity, considering only excitatory probabilistic neurons, it is highly suitable for our goals. Briefly, the network has *N* excitable elements, where each element, *i*, has *n* states: *s*_*i*_ = 0 is the resting state, *s*_*i*_ = 1 is the excited state, and the remaining states, *s*_*i*_ = 2,3,…,*n*−1, are refractory states. The *i*th element can reach the state *s*_*i*_ = 1 from *s*_*i*_ = 0 in two different ways; (1) because of an external stimulus provided by a Poisson process with rate *r* (transition probability *λ* = 1−exp(−*r*Δ*t*) per time step, Δ*t* = 1 ms), or (2) with probability *p*_*ij*_ because of a neighbor *j* being in the state *s*_*j*_ = 1 in the previous time step. The dynamics after excitation are deterministic, i.e., after *s*_*i*_ = 1, in the next step it will change to *s*_*i*_ = 2, and this will continue to occur until state *s*_*i*_ = *n*−1 leads to the *s*_*i*_ = 0 resting state, forming a cyclic cellular automaton. We employed an Erdös–Rényi undirected random network, with *NK*/2 links assigned to randomly chosen pairs of elements. Therefore, we obtain a network with average connectivity *K*, where each element *i* = 1,2…,*N* is randomly connected to *K*_*i*_ neighbors. The probability *p*_*ij*_ of an element *j* activates another element *i* is given by a random variable with uniform distribution in the interval [0,*p*_max_]. The local branching ratio is given by $\sigma_{j}=\sum_{i}^{K_{j}}p_{ij}$ and corresponds to the average number of excitations generated in the next time step by the *j*th element. The average branching ratio *σ* = ⟨*σ*_*i*_⟩ is the parameter that sets the criticality (*σ* = 1), which is chosen using *p*_max_ = 2*σ*/*K*.

The average activity *F* is defined as $F=T^{-1}\sum_{t=1}^{T}\rho_{t}$, where *ρ*_*t*_ is the network instantaneous density activity of elements *s* = 1 at time *t* and *T* is a large time window (of the order of 10^3^ ms) [1]. The response curve is defined as the average activity, *F*, dependent on the stimulus rate *r*. The network has a minimum response, *F*_0_, and a maximum response, *F*_max_. The dynamic range is defined as Δ=10log(r0.9r0.1), in dB, as the interval whose variations in the stimulus result in robust variations of *F*. The interval [*r*_0.1_, *r*_0.9_] is found from the correspondent interval [*F*_0.1_, *F*_0.9_], where *F*_*x*_ = *F*_0_ + *x*(*F*_max_−*F*_0_) [1].

The avalanches were triggered by the random activation of one single element and the spontaneous activity was recorded till the active elements became absent. The avalanche size was defined as the amount of involved active elements without considering repetitions. This procedure was repeated *n*_ava_ times to generate the probability density function (PDF) of avalanche sizes. We also calculated the linear least squares regression to check the power law exponent and the R-squared coefficient.

### Spatiotemporal activity entropy evaluation

To evaluate the entropy on the basis of the activity of the networks, 10% of the elements were randomly activated at the initial time. First, as an indication of the homogeneity of individual element activity during *n*_ava_ avalanches, we counted the number of times each element was activated (*s* = 1). Thus, we were able to obtain the activation PDF of element *i*, *F*_S_(*i*). If the activity between the elements was homogeneously distributed, a flat distribution would have been observed. Therefore, we used a theoretical curve of a constant PDF *F*_*C*_(*i*) as reference. We defined the parameter Δ*d* as a measure of the distance between the PDF obtained by simulation in relation to the theoretical one:

$$\Delta d=\frac{1}{N}\sum_{i=1}^{N}\left|F_{C}(i)-F_{S}(i)\right|.$$

The greater the value of Δ*d* was, lesser was the homogeneity of the resulting activity.

**Fig 1:**
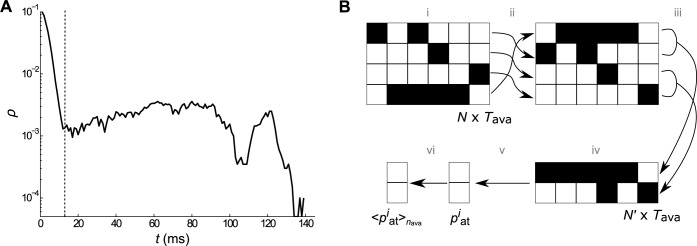
Reduction method employed in the analysis of the network activity. Method used to obtain a smaller network, which resembled the same features of activation probability density function *F*_S_ as those of the original. (A) Example of the network density activity *ρ* during the *j*th avalanche. The time window $T_{\mathrm{ava}}^{j}$ starts at approximately 13 ms. (B) Simplified representation of the reduction method. The first step was the arrangement of the element activities during an avalanche in a matrix of size $N\times T_{\mathrm{ava}}^{j}$ (step i), followed by ordering the elements according to their activity (step ii). The dimension of the matrix was reduced by a factor of *c* = 1250, grouping sequential elements (step iii), and each new element was considered active if at least 2 of its components was activated (step iv). Then, the probability of activation of each element was calculated by time, ${p_{a}^{i}}_{t}$ (step v), and finally the mean from *n*_ava_ = 10 000 avalanches, ${\langle {p_{a}^{i}}_{t}\rangle }_{n_{\mathrm{ava}}}$, was calculated (step vi).

In order to calculate the activity entropy, we reduced the random networks to full connected smaller ones of size *N*′. The reduction was obtained by following these steps, as shown in Fig 1B: (i) *n*_ava_ avalanches were simulated with the time window $T_{\mathrm{ava}}^{j}$ (explained later), with *j* = 1,2,…,*n*_ava_; (ii) the elements of the networks were set in an order in a matrix of $N\times T_{\mathrm{ava}}^{j}$ for each avalanche on the basis of their activity; (iii) the dimension of the matrix was reduced by a factor *c*, where *N*′ = *N*/*c*, thereby grouping sequential elements; (iv) each new element was considered active if *n*_act_ amount of its components were activated; (v) the probability of activation of each element with time, ${p_{a}^{i}}_{t}$, was calculated for each new element, with *i* = 1,2,…,*N*; (vi) the mean probability by avalanche ${\langle {p_{a}^{i}}_{t}\rangle }_{n_{\mathrm{ava}}}$ was calculated. The time window $T_{\mathrm{ava}}^{j}$ of the *j*th avalanche was shown in Fig 1A. It was defined as the interval that corresponded to the period after the initial high activity provoked by the external stimulus and after the final drop. *T*_ava_ was the longest of all $T_{\mathrm{ava}}^{j}$, i.e., $T_{\mathrm{ava}}=\max\{T_{\mathrm{ava}}^{j}|j=1,2,\ldots ,n_{\mathrm{ava}}\}$.

After determining ${\langle {p_{a}^{i}}_{t}\rangle }_{n_{\mathrm{ava}}}$ for each element *i*, we applied a minimalistic model for the network dynamic. In this model, the elements had two states, active and inactive, indicated by 1 and 0, respectively. The model started with one randomly chosen single active element. For each step, the active nodes in the previous step became 0 and remain 0 for one more time step, the nodes *i* that had active neighbours *j* in the previous step have the probability $p_{ji}^{\prime }$ to become active.

In order to obtain the news $p_{ij}^{\prime }$ for the reduced networks, we normalized ${\langle {p_{a}^{i}}_{t}\rangle }_{n_{\mathrm{ava}}}$ by its mean value and obtained one variable similar to the *σ*_*i*_, defined as *γ*_*i*_. So, the $p_{ji}^{\prime }=\frac{\gamma_{i}}{N^{\prime }}$, with $p_{ij}^{\prime }\neq p_{ji}^{\prime }$ and $\gamma_{i}=\sum_{j=1}^{N^{\prime }}p_{ji}^{\prime }$. While *σ*_*i*_ is related to the number of activation that departs from element *i*, *λ*_*i*_ is related to the number of possible neighbours that are able to activate the element *i*.

The activity entropy *H*_A_ was based on the spatiotemporal patterns of the avalanches. For each time step, we represented the activity by a vector of size 1 × *N*′. An avalanche of length *t*′ was created by concatenating *t*′ vectors into a single one of size 1 × *N*′*t*′ [2]. These vectors were then tested using a similarity matrix. For the original networks the length *t*′ was chosen by setting *t*′ = *T*_ava_, and for the reduced networks we considered the same time window for all the avalanches. We did not take into account the initial time, and by doing this we did not consider the first random active element in each avalanche.

The similarity between two patterns varied from 0 to 1, and is defined as [11]:

$$\mathrm{Sim}(v_{i},v_{j})=\frac{\langle v_{i},v_{j}\rangle }{\langle v_{i},v_{i}\rangle +\langle v_{j},v_{j}\rangle -\langle v_{i},v_{j}\rangle },$$

where ⟨∙, ∙⟩ is a dot product and *v* are the vectors of configurations. Finally, the entropy *H*_A_, in bits, was defined as *H*_A_ = −∑_*i*_*p*_*i*_log_2_(*p*_*i*_), where *p*_*i*_ is the probability of the *i*th activity configuration considering the total patterns found when Sim ≥ *th*, with *th* is an arbitrary threshold.

## Results

### Networks with different connectivities produce similar dynamic features

**Fig 2:**
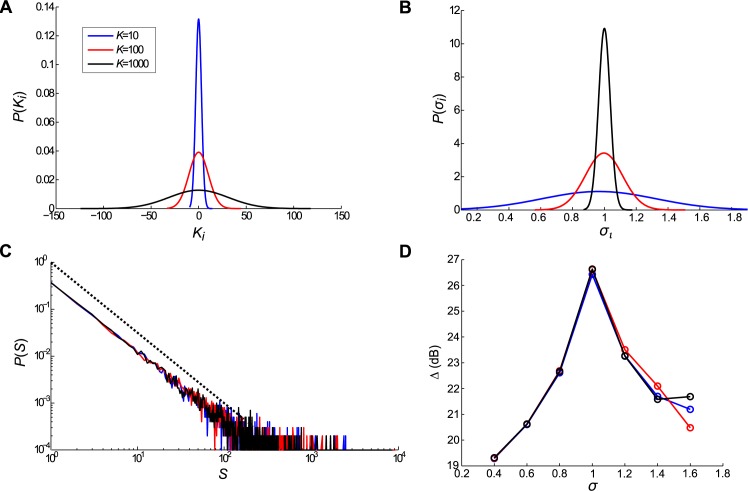
General features of the networks and activity. In our network model, the total number of elements was *N* = 20 000 and we used 3 distinct values for mean connectivity; *K* = 10, 100, and 1 000. We simulated *n*_ava_ = 10 000 avalanches triggered by one random active element in the initial time with a dynamic model based on [1], as explained in the Methods section. (A) Probability density distributions of connectives *K*_*i*_ *P*(*K*_*i*_) for *K* = 10 (red), 100 (blue), and 1 000 (black) centralized in 0, that is, *K*_*i*_−*K*. (B) Probability density distributions of the branching parameters *σ*_*i*_ *P*(*σ*_*i*_) for *K* = 10 (red), 100 (blue), and 1 000 (black). (C) Probability density distributions of the avalanche sizes *P*(*S*) for *K* = 10 (red), 100 (blue), and 1 000 (black). The dashed black line has a reference slope of −1.5. (D) Dynamic range Δ *versus σ* for networks with *K* = 10 (red), 100 (blue), and 1 000 (black).

We started simulating avalanches in critical networks using the methodology described before. We generated networks with the same number of nodes but distinct mean connectivities *K*. The simulations were performed using 3 chosen values of *K*, *K* = 10, 100, and 1 000, with *N* = 20 000. Fig 2A and 2B show the *K*_*i*_ and branching parameter *σ*_*i*_ distributions for the three cases. The *K*_*i*_ distributions were centralized in 0, that is, *K*_*i*_ − *K*. Because the inverse dependence between the *p*_max_ and *K* parameters, as the *P*(*K*_*i*_) became more broad, the *P*(*σ*_*i*_) became more sharp. We used *σ* as the control parameter because of the randomness of the *p*_*ij*_. Otherwise, it is known that the best parameter control is given by the largest eigenvalue of the connection matrix [29].

Fig 2C show the avalanche size distributions calculated for *n*_ava_ = 10 000, where the dashed line is a curve with a referent slope of −1.5. The slopes were calculated using linear least squares regression, which provided the fitted exponent values of −1.48 (−1.55, −1.41), −1.47 (−1.54, −1.41), and −1.45 (−1.52, −1.39) for *K* = 10, 100, and 1 000, respectively, with R-square value of 0.99 for all fits. The dynamic range responses related to *σ* were also calculated (Fig 2D) showing similar values for all networks. Although the networks have different *K* and distributions of *K*_*i*_ and *σ*_*i*_, all three exhibited very similar avalanche statistics as revealed by the power law fitting, including similar dynamic responses.

### Homogeneity of activity in network elements depends on mean connectivity

**Fig 3:**
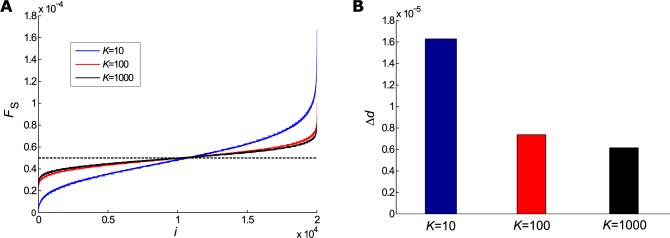
Analysis of individual element activity. The activity of each element *i* of the networks during the avalanches was computed and activation probability density function (PDF) of elements *i F*_S_ was calculated. We simulated *n*_ava_ = 10 000 triggered by random 10% active initial elements. (A) We simulated activity in 10 distinct networks for each mean connectivity, *K* = 10 (red), 100 (blue) and 1 000 (black). The dashed black line represents homogeneity reference PDF, *F*_C_. All 10 simulations were plotted in the graph and appeared as overlapped lines. (B) We also calculated the mean difference Δ*d* index between the results obtained for each condition and those obtained for the reference line.

As we were able to determine that networks with distinct mean connectivities produce similar global dynamics, we next investigated the individual activity during the avalanches. Therefore, we analysed the activation PDF, *F*_S_, of the element *i* and the homogeneity index, Δ*d*, for the above-mentioned 3 cases with *n*_ava_ = 10 000, *N* = 20 000, and 10% of active initial elements (Fig 3). Fig 3A shows *F*_S_ for *K* = 10, 100 and, 1 000, with *F*_C_ as the homogeneity reference. The overlapped lines represent results from 10 networks of each case. Fig 3B shows the results of mean, Δ*d*, and the errors obtained, i.e., (16.300 ± 0.060) ∙ 10^−6^, (7.400 ± 0.014) ∙ 10^−6^, and (6.150 ± 0.005) ∙ 10^−6^ for *K* = 10, 100, and 1 000, respectively. Our simulations revealed that homogeneity of activity in network elements depended on the mean connectivity. Indeed, higher values of *K* resulted in smaller Δ*d* values. As shown in Fig 2A and 2B, smaller *K* implies a broad *σ*_*i*_ distribution, leading to a higher variety of *σ*_*i*_ values. The largest values could produce more probably paths, in contrast to the smallest ones. That way, these results revealed that homogeneity in the involvement of individual elements in the network activity varied depending on the mean connectivity, although the global features were very similar.

### Network reduction method maintains similar activation features of the original networks

**Fig 4:**
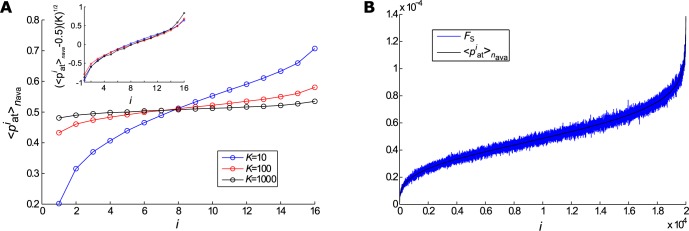
The probability of activation of the reduced networks *versus* the original networks. The reduction methods generated the activation probability for the reduced networks, and keep the tendency with the *F*_S_. (A) The activation probabilities ${\langle {p_{a}^{i}}_{t}\rangle }_{n_{\mathrm{ava}}}$ of the reduced networks for the three cases, *K* = 10 (red), 100 (blue) and 1 000 (black). The inset shows the collapsed curves adopting $({\langle {p_{a}^{i}}_{t}\rangle }_{n_{\mathrm{ava}}}-0.5){\left(K\right)}^{1/2}$. (B) Example of the tendency between the ${\langle {p_{a}^{i}}_{t}\rangle }_{n_{\mathrm{ava}}}$ and the *F*_S_ for the original network with *K* = 10. Both were normalized by the area under the curve.

Networks made up with different configurations, although had showed similar avalanches features and dynamic responses, could hidden features that could only be seen if one had looked closer. In order to analyse the spatiotemporal patterns of the activity in these networks with more details, we employed a reduction method. The reduction decreased the number of possible paths facilitating the analyses, besides ensures the same features of *F*_S_ as those of the original simulated networks. Moreover, in reduced networks we were able to maintain the same *K*, as *K* = *N*′ − 1 = 15. (Fig 1). First, we chose the time window $T_{\mathrm{ava}}^{j}$ as the interval corresponding to the period after the initial high activity provoked by the external stimulus and after the final drop (Fig 1A). In our analysis we computed spatiotemporal patterns after 13 ms to avoid the background activity related to the random stimulus and *T*_ava_ = 413,462, and 273 ms for *K* = 10, 100, and 1 000, respectively. We then arranged the avalanche into a matrix of size $N\times T_{\mathrm{ava}}^{j}$ (step i) and ordered it by activity (step ii). The dimension of the matrix was reduced by a factor of *c* = 1 250, grouping sequential elements (step iii). Each new element was considered active if at least *n*_act_ = 2 of its components was activated (step iv) (Fig 1B). Then, we computed the probability of activation of each element with time ${p_{a}^{i}}_{t}$ (step v) and finally the mean from *n*_ava_ = 10 000 avalanches ${\langle {p_{a}^{i}}_{t}\rangle }_{n_{\mathrm{ava}}}$ (step vi). The values obtained for ${\langle {p_{a}^{i}}_{t}\rangle }_{n_{\mathrm{ava}}}$ are shown in Fig 4A. These probabilities were used in the reduced networks, as explained in the next section. It is noted that the curves collapsed themselves when the transform $({\langle {p_{a}^{i}}_{t}\rangle }_{n_{\mathrm{ava}}}-0.5){\left(K\right)}^{1/2}$ is used. Results were shown as inset in Fig 4A. We observed that the same dependence with *K* was valid for the *F*_*S*_.

There is a little difference between calculation of *F*_*S*_ and ${\langle {p_{a}^{i}}_{t}\rangle }_{n_{\mathrm{ava}}}$, but they follow the same general tendency as we can see for the original network with *K* = 10 as example (Fig 4B). That way, the *F*_*S*_ of the simulations of the reduced networks could be calculated to check the method functionality.

### Entropy as coded by spatiotemporal patterns depends on mean connectivity

The capacity of a network stores information is related to the nodes that are activated, for example, in a neuronal network. Not only the amount of nodes, but the temporal sequence of activation, and if the same sequence could be posteriorly achieved again. A highly used way to characterize the distribution of these patterns is based on the Shannon entropy, and can give us some clues of how the entropy can vary in critical networks.

**Fig 5:**
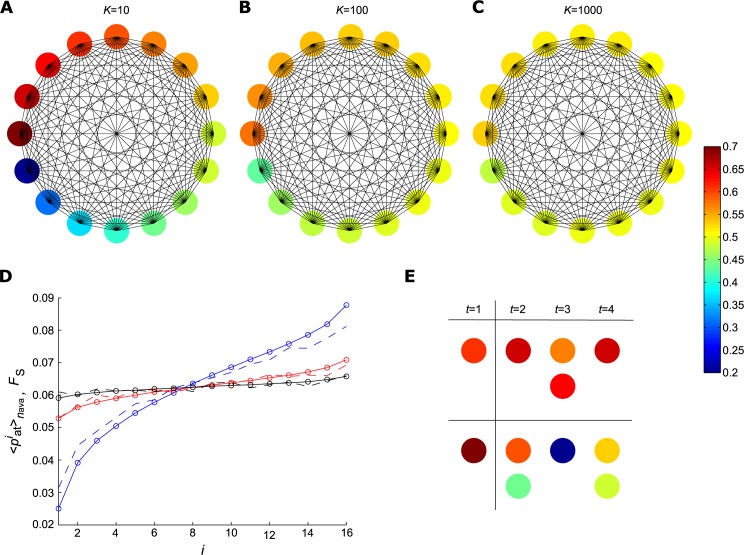
Reduced networks. Scheme of the ${\langle {p_{a}^{i}}_{t}\rangle }_{n_{\mathrm{ava}}}$ for the reduced networks. (A), (B) and (C) show the activation probabilities ${\langle {p_{a}^{i}}_{t}\rangle }_{n_{\mathrm{ava}}}$ coming from the original three cases, *K* = 10, 100 and 1 000 respectively, distributed in fully connected reduced networks of size *N*′ = 16. (D) show the comparison between ${\langle {p_{a}^{i}}_{t}\rangle }_{n_{\mathrm{ava}}}$ obtained from the original networks, circles, and the *F*_S_ calculated from the simulations of the reduced networks, dashed lines, both normalized by the area under the curve. The colours blue, red and black are related to *K* = 10, 100 and 1 000 respectively. (E) Example of patterns observed in networks with *K* = 10. The pattern that was observed more frequently is located in the top, whereas the pattern that was observed less frequently is located in the bottom. The vertical line separates the initial time, when the active element was chosen randomly.

To evaluate the entropy based on spatiotemporal patterns we used the reduced-sized networks for our simulations from now on. Then, each one of the original networks generated a reduced one, which are shown in Fig 5A, 5B and 5C. They are fully connected and the nodes activation probability are featured by the colour distribution. For the simulations we chose *N*′ = 16, *t*′ = 4 ms and *n*_ava_ = 10 000. Calculating the *F*_S_, the results are shown in Fig 5D, from which we can see the agreement between the values obtained from reduced and original networks, both normalized by the area under the curve.

**Fig 6:**
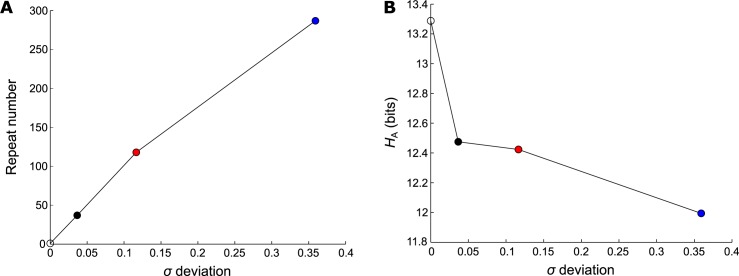
Analyses of the entropy as the result of spatiotemporal patterns. A minimalistic model was applied for the dynamics in the reduced networks with size *N*′ = 16, activation probabilities ${\langle {p_{a}^{i}}_{t}\rangle }_{n_{\mathrm{ava}}}$, *n*_ava_ = 10 000 and *t*′ = 5 ms, as described in Methods section. The avalanches were compared using similarity matrix, and were subjected to an arbitrary threshold *th* = 0.7 in order to identify the similar patterns. The initial activated elements were not take into account. After this, we identified the number of different patterns and the maximum repeatability founded in each case. (A) The maximum repeatability of each case *versus* the *σ*_*i*_ deviation. (B) The entropy *H*_A_ *versus* the *σ*_*i*_ deviation. The associated *K* with the *σ*_*i*_ deviation is colour highlighted. Blue, red and black are related to *K* = 10, 100 and 1 000 respectively. The colourless circle is associated to the theoretical result where repetition is not observed.

The simulated avalanches generated 10 000 vectors of activity of size 1 × *N*′*t*′. Based on these vectors, we calculated the similarity matrix. Applying the threshold *Th* = 0.7, all values above it were considerated as 1 and below it as 0. That way, we identified all similar patterns and computed the activity entropy *H*_A_. Fig 5E shows one example for *K* = 10 related to patterns that repeated more frequently (top), as well as patterns that repeated less frequently (bottom). We can see that the less repeated patterns were generally composed by the elements with low activation probability, in contrast with the more repeated patterns. The vertical line separates the initial time, when the active element was chosen randomly. Fig 6A shows that maximum repeatability of the patterns and Fig 6B the *H*_A_ *versus* the deviation of the *σ*_*i*_ distribution. The markers colour are related to the referent *K* and the colourless marker are the theoretical value idealized for a network with 10 000 avalanches and 0 patterns repeated. We can see that the repeatability increases as the deviation increases (or the *K* decreases) and the *H*_A_ has the opposite behaviour.

We were able to determine that the entropy increased monotonically with *K*, reinforcing the results observed in Fig 3. Indeed, the most likely spatiotemporal patterns increased the probability of repetition and decreased the entropy. In contrast, an increase in *K* produced more equi-probable spatiotemporal patterns, leading to an increase in entropy.

## Discussion

Critical dynamics have been considered as an optimum regime for neuronal networks, mainly to enhance both information processing and dynamic range. Despite the importance of the criticality, the knowledge about embedding information in the phase transition provided by this regime is very limited.

In this study, we employed branching process based models to study the spatiotemporal activity entropy. Our results revealed that the information capacity of networks set within the limits of critical dynamics may vary depending on the choice of parameters. Distinct values of *K* resulted in different distributions of *σ* with equal means but decreasing standard deviations. A large deviation in *σ* led the networks to superimpose some paths that repeated more than others, even creating a large number of patterns such as those fitted to a power law distribution.

The existence of more likely paths induced a reduction in the entropy as coded by spatiotemporal patterns. Therefore, it was possible to obtain variations in the information capacity even inside the critical regime, which does not interfere with both input sensitivity and dynamic response range. Moreover, adaptation in dynamic range response has been investigated as a physiological mechanism in neuronal cortical networks [30].

Although the model employed here has specific features, such as the dependence of *K* and *σ* variables, our results can be extended to neuronal biology. In this context, the synaptic plasticity plays a fundamental role, increasing or decreasing the recurrence of certain connections, producing more or less likely spatiotemporal activity paths. We can speculate that this balance is required in a neuronal network. For example, in circuitries related to memory and learning, spatiotemporal patterns may repeat more frequently than expected by chance. In this condition, neuronal circuitries do not work in full capacity in relation to the information, although this particular feature is maximum in critical regime.

Others models have been used to examine the repetitive patterns found in neuronal activities, such as the spike-timing dependent plasticity (STDP)-based learning process [13,31], where phase coded spike patterns are stored in the synaptic connections. Another example is related to learning model presented by de Arcangelis *et al*. [32]. In that paper the authors showed a network model with plastic synaptic strength that presented critical behaviour and also was able to learn. They concluded that the system learns more efficiently as more possible spatiotemporal paths exist. However, in a simpler version, branching process models can achieve repeatable patterns, more or less, changing the deviation of the branching parameter, which may also result in controlling of information capacity.

To summarize, our findings disclosed new theoretical predictions involving dynamics and topology in real neuronal networks. For example, it was possible to postulate that brain circuitries located in the cortex might comprise a large amount of information as encoded by numerous distinct spatiotemporal patterns [6]. In contrast, we demonstrated that other circuitries may show a dynamic response, but with less information entropy. For example, when compared with those of the cortex, neuronal circuitries in the brain stem might not require a massive combination of distinct spatiotemporal patterns, although circuitries should be able to maintain optimum sensitivity to stimulus. Notably, a large variety of neuronal subtypes in the brain stem have been described [33,34]. The role of morphological heterogeneity, which may underlie variations in the connectivity degree, in the formation of networks with decreased entropy is a matter that needs to be empirically investigated.
